# Connectomic stroke lesion measures provide no benefit over basic spatial lesion features in the prognosis of global stroke outcome measures

**DOI:** 10.1093/braincomms/fcaf268

**Published:** 2025-07-28

**Authors:** Christoph Sperber, Laura Gallucci, Vanessa Kasties, Marcel Arnold, Roza M Umarova

**Affiliations:** Department of Neurology, Inselspital, University Hospital Bern, University of Bern, Bern 3010, Switzerland; Department of Neurology, Inselspital, University Hospital Bern, University of Bern, Bern 3010, Switzerland; Child Development Center, University Children’s Hospital Zurich, University of Zürich, Zürich 8008, Switzerland; Department of Neurology, Inselspital, University Hospital Bern, University of Bern, Bern 3010, Switzerland; Department of Neurology, Inselspital, University Hospital Bern, University of Bern, Bern 3010, Switzerland

**Keywords:** prediction, disconnection, white matter, graph, brain mode

## Abstract

The prediction of stroke outcome from imaging markers could be used to guide individualized therapeutic approaches. We aimed to find the best imaging marker to predict the global functional impact of a stroke lesion among low- to high-level connectomic measures—indirect estimations of structural connectivity, graph representations, or brain modes—as well as spatial lesion features. This observational study retrospectively analysed clinical routine data from patients with acute first-ever ischaemic stroke. We traced lesions in diffusion-weighted MRI and computed 21 topographic or connectomic measures, including (i) tract-wise, voxel-wise and interregional white matter disconnection that were indirectly estimated by reference to healthy connectome data; (ii) interregional network structure by graph measures; and (iii) brain modes, which represent elementary interactions between grey matter regions. We used all features to predict stroke severity [National Institutes of Health Stroke Scale (NIHSS) 24 h] or classify poor functional outcome (mRS 3 months ≥ 2) in a nested cross-validation with high-dimensional machine-learning models. For comparison to specific, granular post-stroke cognitive deficits, we replicated the modelling procedures in another sample for selective attention and phonemic word fluency. The study included 755 patients [mean age = 66.9 ± 15.3 years; NIHSS 24 h median (IQR) = 2 (1; 5); mRS 3 months = 1 (0; 2)]. For both measures, simple spatial lesion features (NIHSS 24 h: *R*² = 0.395 ± 0.059; mRS: accuracy = 65.62% ± 3.45, positive predictive value = 0.72 ± 0.13; negative predictive value = 0.64 ± 0.04) outperformed connectomic measures (all *P* < 0.0007), even though the predictions of the best measures in each category were numerically close. Control analyses on specific cognitive deficits in a sample of 182 patients found connectomic measures to be equal or even superior to spatial lesion features. Connectomic stroke imaging markers provide no benefit in the prediction of acute stroke severity and functional outcome at 3 months. Spatial lesion imaging features seem to effectively capture the global neurological perturbation caused by a stroke lesion and could provide a basis for personalized prediction algorithms. On the other hand, connectomic stroke imaging markers may be warranted when modelling specific post-stroke cognitive deficits.

## Teaser

Sperber *et al*. report that indirectly estimated connectomic stroke imaging markers do not enhance the prediction of acute stroke severity and functional outcome at 3 months. Spatial lesion imaging features seem to effectively capture the global neurological perturbation caused and could provide a basis for personalized prediction algorithms.

## Graphical Abstract

**Graphical Abstract:**
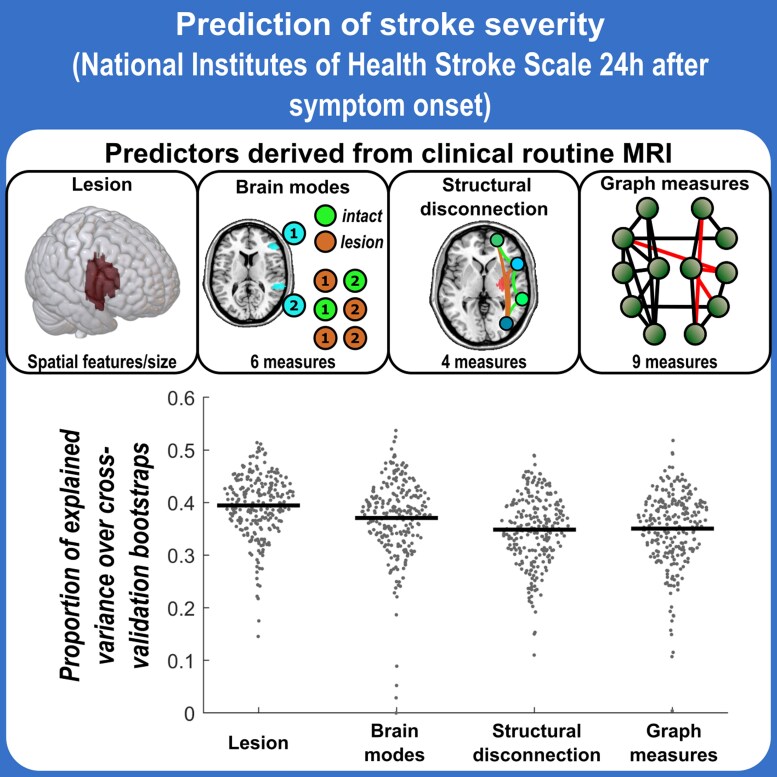

## Introduction

Acute stroke therapy has made tremendous progress in recent years, and it depends critically on the availability of brain imaging.^1,2^ Although imaging is an integral part of any clinical protocol in the treatment of acute stroke, brain imaging is not systematically used to make predictions about functional outcomes and to guide long-term individualized therapy. While the objective of utilizing data-driven computational methods for this purpose was formulated years ago,^3,4^ its implementation in clinical practice is still pending. On top, there is still no consensus on how to conceptualize a brain lesion—an issue that traces back to our fundamental understanding of anatomo-functional brain architecture.

This conceptual disagreement is evident given the many competing approaches to predict stroke outcomes based on a structural lesion visible in brain imaging. The most straightforward approach directly represents the lesion by its location as the voxel- or region-wise lesion status.^5,6^ Driven by innovations in fibre bundle imaging and the generation of high-resolution reference connectomes of healthy brains, the indirect estimation of white matter disconnection became widely used.^7-11^ However, white matter disconnection has been represented in many different, apparently equivalently meaningful, ways, such as fibre bundle lesion load, disconnection of major fibre bundles and interregional disconnection, and by projection into voxel-wise maps.^12-19^ Moreover, some studies used directly or indirectly estimated disconnection as a foundation for computing graph measures of lesion-induced damage.^13,20-22^ The large family of graph measures can represent the higher-level structure of a network that composed of nodes (here: brain regions) and edges (here: white matter connections between regions). They quantify various aspects of a network from a local to a global level. On a local level, graph measures can, e.g. represent how strongly connected a brain region is and, thereby, identify network hubs. On a global level, they represent general network aspects, such as how efficiently or densely a network is connected.^23^ Graph measures are widely used to represent neural network structure in neurology^24-26^ and were suggested to allow a generally deeper mechanistic understanding of brain pathology.^27,28^

A different perspective on the brain is taken by studies that assume a central role of brain modes.^29,30^ These focus on the coalitional status of grey matter structures and, thereby, represent different low-level configurations of how the functionality of brain regions can be associated within a network. They represent how brain regions interact with each other in providing the basis for neural and cognitive processes, for example, in a complementary additive, an antagonistic inhibitory or a functionally overlapping manner. The assumption of brain modes was suggested to explain the emergence of functional deficits after specific lesion patterns. For example, brain modes can explain when lesions to separate regions evoke the same deficit, or when paradoxical remission effects emerge after recurrent damage to different brain regions.^30,31^

Numerous conceptually very different ways to represent brain pathology induced by a focal brain lesion were suggested.^32-36^ While this plurality of representations leads to obvious fundamental questions about our theories of the human brain, it also leads to the more practical question of how to conceptually and mathematically represent a lesion when we aim to predict stroke outcomes from stroke imaging. A connectomic data representation might predict global stroke outcome if it better represents the neural correlates of the accumulated sub-deficits that strongly impact global stroke outcome.

In the present study, we sought to find the best imaging marker to predict the functional impact of a stroke lesion among the many available lesion representations. We compared the predictive value of 21 different lesion imaging marker sets, including simple spatial lesion information, indirectly estimated disconnection measures, derived graph-based disconnection measures, and brain modes in high-dimensional machine-learning-based prediction models.

## Materials and methods

### Patients and clinical assessment

In a retrospective observational study, we analysed clinical routine data of stroke patients admitted to the Bern Stroke Centre between January 2015 and October 2020. Inclusion criteria were (i) a first-ever ischaemic stroke in the anterior circulation; (ii) availability of MRI acquired ∼24 h after admission; and (iii) no existing records of pre-stroke disability [modified Rankin Scale (mRS) > 1]. We excluded patients who had a second stroke within 3 months because such would have confounded the association between acute imaging markers and longer-term stroke outcomes. A flowchart in Supplementary Fig. 1 documents the recruiting process and all exclusions.

Acute stroke severity was assessed by the attending physician using the NIHSS 24 h after stroke onset. The study focus was the functional impact of stroke and not acute hypoperfusion or reperfusion therapy. Hence, we analysed the NIHSS at 24 h and not at admission, as this measure better reflects the functional impact of the final lesion in patients who undergo acute therapy. Functional outcome at 3 months after stroke onset was assessed with the mRS at follow-up by the attending physician or by phone. All participants or their guardians gave written informed institutional general consent for research. The study received approval from the local ethical board (Kantonale Ethikkommission Bern KEK 2020-02273).

### Brain imaging and lesion segmentation

Lesion segmentation was performed on clinical routine MR images acquired with a 1.5 T or 3 T Siemens scanner ∼24 h after symptom onset. We demarcated the lesion area with a semiautomatic algorithm in SPM12 (http://www.fil.ion.ucl.ac.uk/spm/software/spm12) on diffusion-weighted images with a slice thickness of 4–5 mm as described previously.^37^ The semiautomatic algorithm required the manual selection of intensity thresholds to optimally match the lesion mask and the hyperintense area of diffusion-restricted brain tissues. Other available sequences were consulted to guide the procedure and verify accuracy. The binary lesion masks were then warped to the Montreal Neurological Institute space at 2 × 2 × 2 mm³ with the normalization parameters of the co-registered T1 scan. These procedures were performed and checked by a neurologist with over a decade of research experience with lesion mapping (R.M.U.). Lesion size was defined as the volumetric size of the normalized lesion map. Both the voxel-wise ‘spatial lesion’ ‘features’ defined by the binary lesion masks and ‘lesion size’ were used as predictors.

### Estimation of white matter disconnection

We estimated the disconnection of white matter fibres by reference to the HCP-842 population-averaged streamline atlas^38^ with the Lesion Quantification Toolkit.^15^ Such indirect estimation of white matter disconnection identifies all white matter streamlines that are expected to be disconnected by a focal lesion and, hence, can estimate a stroke patient’s disconnectome without the requirement of running dedicated diffusion tensor MRI sequences and performing fibre tracking. This well-established method^12,14-16^ has previously provided outcome markers for a multitude of neurological deficits.^8,10,11^

Lesion masks were resliced to the default imaging space of the Lesion Quantification Toolkit. For each patient, the toolbox identified all streamlines in the HCP-842 streamline atlas that intersect the lesion area defined by the normalized lesion map and, based on this data, generated several disconnection measures. All processes that involved a parcellation of grey matter areas used the BN-246 human Brainnetome atlas.^39^

### Disconnection measures

**Figure 1:**
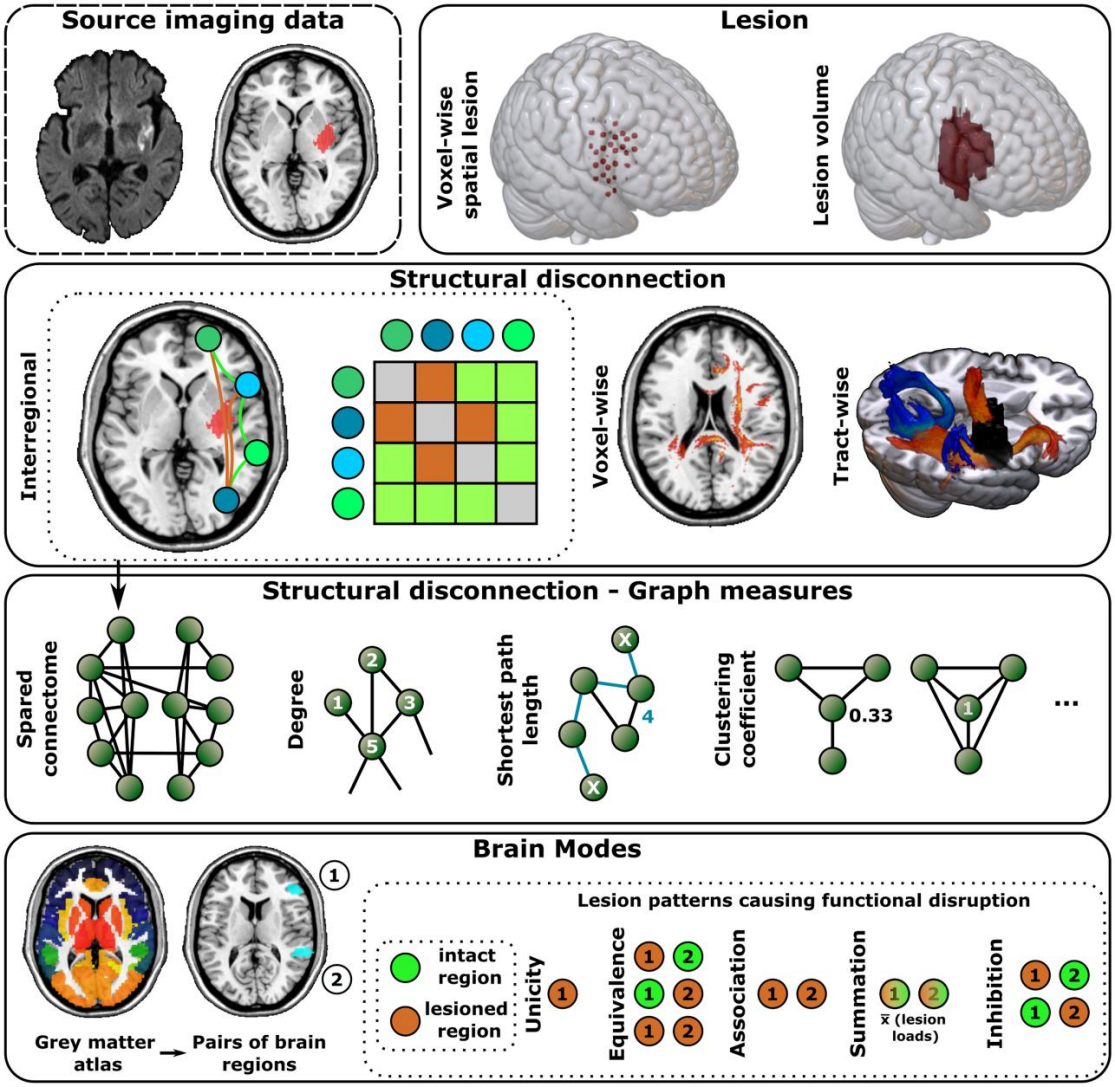
**Schematic overview of lesion imaging and connectomic measures.** Non-exhaustive overview of included topographic or connectomic features.

The output of the Lesion Quantification Toolkit included multiple representations of structural disconnection (Fig. 1). All measures are accessibly described in the accompanying publication.^15^ ‘Tract-wise disconnection’ represented the proportion of disconnected streamlines across 70 canonical white matter tracts (e.g. the corticospinal tract and superior longitudinal fasciculus) found within the reference streamline atlas.^38^ ‘Disconnection maps’ represented the disconnection on a voxel-wise level. In each voxel, all passing streamlines were identified, and the proportion of disconnected streamlines was mapped. The ‘interregional disconnectome’ represented the disconnection between all grey matter areas in the BN-246 parcellation. Herein, all streamlines that connected the given two regions were identified, and the proportion of disconnected streamlines was calculated.

Based on the interregional disconnectome, we further created a spared ‘binary interregional connectome’. This feature indicated the spared interregional connections in each patient, i.e. for existing interregional connection, it indicated if it was intact (‘1’) or disconnected (‘0’). As only a minority of all pairs out of the 246 regions are connected in the healthy brain, we first identified all region pairs connected by at least one streamline in the HCP-842 atlas to define a healthy reference connectome. Next, we defined a cut-off at which the proportion of disrupted interregional streamlines constitutes relevant disconnection. While such a cut-off has previously been chosen arbitrarily,^20^ we performed a sub-analysis to maximize the within-sample association of binarily defined disconnection and stroke severity for cut-offs from 5% to 95% in 5% steps across all connections (see Supplementary section Optimization of binary cut-off for interregional disconnection and Supplementary Fig. 2). We found that a 90% cut-off maximized the disconnection–stroke severity associations. We used this cut-off of at least 90% of disrupted streamlines to identify disconnected region pairs. For each patient, we then computed the spared ‘binary interregional connectome’ by subtracting the matrix identifying the patient’s disconnected region pairs from the healthy reference connectome. It was used as another predictor variable and served as a starting point for the computation of graph measures, which required a binary matrix structure.

### Graph representations of disconnection

Based on the spared binary interregional connectome, we computed several graph measures to describe the spared structural network (Fig. 1). We computed both local and global graph network metrics using the Brain Connectivity Toolbox.^22^ We considered all region pairs with at least one streamline in between in the healthy HCP-842 atlas as connected. The measures represent the network structure given the network nodes—here, the brain regions defined by the BN-246 atlas—and the edges between—here, the spared white matter connections between each pair of nodes. For most graph measures, we assessed the difference between the patient’s spared connectome graph structure and the healthy connectome graph structure in the HCP-842, similar to previous studies that investigated the difference in the shortest structural path length.^15,20^ These variables are denoted by ‘Δvariable’.

The measures included the node-wise ‘Δdegree’, the change in the number of edges connected to a node; node-wise ‘Δclustering coefficient’ describing the change of connectivity of the neighbours of a node; global ‘Δrich club coefficient’, the change in the structure of node degrees in a network; ‘Δdistance’, the change in the length of the shortest path between two nodes; ‘binary Δdistance’, a binarized Δdistance representation that identifies all pairs of regions for which a change in the shortest path length was assumed, independent of the magnitude of this change (as in^20^); ‘global efficiency’, the average inverse shortest path length in the spared network; global ‘characteristic path length’, the average shortest path length of the spared network; node-wise ‘local efficiency’, which applies the concept of global efficiency to the neighbourhood of a node; and node-wise ‘betweenness centrality’, a measure of how many of the spared shortest paths go through a certain node. All graph measures are described in detail in the Brain Connectivity Toolbox paper^22^ and the accompanying homepage (https://sites.google.com/site/bctnet/home). Computational details are reported in the supplementary.

### Assessment of brain modes

Brain modes describe the coalitional state of pairs of brain regions.^30^ We assumed that if brain modes adequately represent the anatomo-functional impact of a lesion, their explicit inclusion in a model could improve the prediction of stroke outcomes, even though the interaction of regions could, to some degree, already be modelled by non-linear prediction algorithms on simple lesion features. Their assessment required a cut-off to define a grey matter area in the BN-246 parcellation as intact or lesioned. For empirical justification, we applied the same strategy as for the definition of intact versus disconnected interregional connections described above. The procedures are described in the supplementary including Supplementary Fig. 3 and suggested to assess brain regions with ≥60% of damaged voxels as lesioned.

We computed brain modes for the 94 regions damaged in at least 10 patients. We first represented ‘unicity’, i.e. the binary status, lesioned or intact, of each single region alone. Although this is considered a brain mode, this variable is conceptually the same as spatial lesion features but with a resolution on the level of regions instead of voxels. All further brain modes apply to pairs of regions—all possible pairs among the 94 regions—and were represented in a separate symmetrical matrix. We identified ‘equivalence’ when at least one of the two regions was damaged and ‘association’ when both regions were affected. Mutual ‘inhibition’ was noted whenever one region, but not both regions, was damaged. ‘Summation’ conceptually slightly deviated from the other brain modes for not being derived from the binary status of a region. Instead, we computed summation as the mean of the lesion load, i.e. the proportion of lesioned voxels, for two regions. Finally, we concatenated the representations of ‘all brain modes’ into a single feature set.

### Prediction modelling and statistical analysis

All data were processed into a format suitable for prediction. Required steps varied and are listed in the supplementary. In general, features with little to no informative variance were removed. Continuous data were normalized to a mean of 0 and a standard deviation of 1; sparse data indicating pathology were standardized between 0 and 1. Redundant features in symmetric matrices were removed. For extremely large-dimensional data (e.g. lesion or disconnection maps), both the raw data and dimensionality-reduced data obtained by principal component analysis were used. For such dimensionality-reduced data, all components explaining >1% of total variance in the original data were retained. Details are listed in Supplementary Table 1.

Prediction models were computed by support vector machines (SVMs), a kernelized, potentially high-dimensional algorithm both for classification and regression using the Regression Learner Toolbox in MATLAB R2023a. We used SVM with a non-linear radial basis function kernel, which we recently have found to be an optimal approach among several suitable prediction algorithms for the given task.^40^ Model hyperparameters—box constraint *C*, kernel scale *γ* and (only in regression) *ɛ*—were optimized by Bayesian optimization within the software’s default ranges (see supplementary for details). All models contained age as an additional predictor. To facilitate regression, the NIHSS 24 h was de-skewed by log_10_(*x* + 1)-transformation. The mRS was binarized into poor (mRS ≥ 2) versus favourable functional outcome (mRS < 2). We chose this commonly used cut-off^41^ as it created relatively equally sized groups, which benefits classification modelling.

The predictive value of each imaging data representation was first assessed by repeated nested cross-validation with model averaging (adapted from Röhrig *et al*.^42^; for illustration^40^). The dataset was split into 5-folds of 151 patients each. In the inner cross-validation loop, 4-folds were used for optimizing model hyperparameters and training. The resulting model was then tested in the fifth, held-out fold to predict the target variable. This procedure was repeated five times, ensuring that each fold served as the held-out test set once. Next, this entire procedure was repeated five times with a pseudo-randomized assignment to folds to control for fluctuations arising from fold assignments. For comparability, the same assignment to folds was used across all data representations. The resulting five predictions per patient were averaged for regression of stroke severity, or the majority decision was recorded for classification of functional outcome. This yielded one single prediction for each patient. The resulting predictions were evaluated for the coefficient of determination *R*² (for regression of stroke severity) or the classification accuracy (for classification of functional outcome).

Second, we augmented the nested cross-validation with a computationally more demanding bootstrapping design to estimate both overall model performance and its variability. For the best-performing data representation in each category (i.e. spatial lesion features, indirect disconnection, graph measures and brain modes) in the previous analyses, we repeated the nested cross-validation 250 times with a varying assignment to folds and, in each iteration, assessed the prediction performance (*R*²/accuracy) in the fifth held-out fold. We compared the prediction performance between the four categories by paired *t*-tests with Bonferroni correction.

### Control analysis: prediction of specific cognitive deficits

To better put the main analyses on the prediction of global measures of stroke outcome into context, we additionally consulted another stroke sample in which we modelled specific post-stroke cognitive deficits—selective attention and phonemic word fluency—using spatial and connectomic lesion imaging measures. The new sample of 182 patients originated from a prospective study on post-stroke cognitive impairment in acute stroke.^43^ Details on the sample are reported elsewhere^43^; details on the measures and the modelling procedure are reported in the supplementary. Due to lower computational demands with smaller samples, we could subject the imaging measures to repeated nested cross-validation with 500 bootstrapped repetitions.

### Sensitivity analyses

We performed sensitivity analyses using the same cross-validation design as in the main analyses. First, we computed a baseline model with clinical and demographic predictors to evaluate the predictive value of non-imaging variables in the classification of functional outcome. We used the variables age, sex, NIHSS 24 h (i.e. a measure of acute baseline deficits), stroke laterality (left/right) and application of acute therapy by intravenous thrombolysis or mechanical thrombectomy (yes/no).

Second, we computed another baseline model with low-dimensional lesion parameters only—lesion laterality (L/R) and lesion size—to predict stroke severity and functional outcome.

Third, we recomputed the functional outcome classification, excluding all patients with mRS = 6, i.e. those who died during follow-up. We recalculated the prediction models using the best imaging feature in each of the four categories. To save computational resources, we used only 50 replications of the cross-validation design.

Fourth, we added sensitivity analyses to evaluate the definition of existing connections in the creation of the ‘binary interregional connectome’. As outlined in section Disconnection measures, every region pair with at least one connecting streamline was considered in the connectome. In the sensitivity analysis, we varied this criterion. The maximum number of streamlines between two regions was 1027. We replicated the prediction of stroke severity and functional outcome with the original criterion *N*_Streamlines_ = 1, but also tested *N*_Streamlines_ = 50, 100 and 350. We evaluated the predictive value of the ‘binary interregional connectome’ with these modified criteria.

## Results

**Figure 2:**
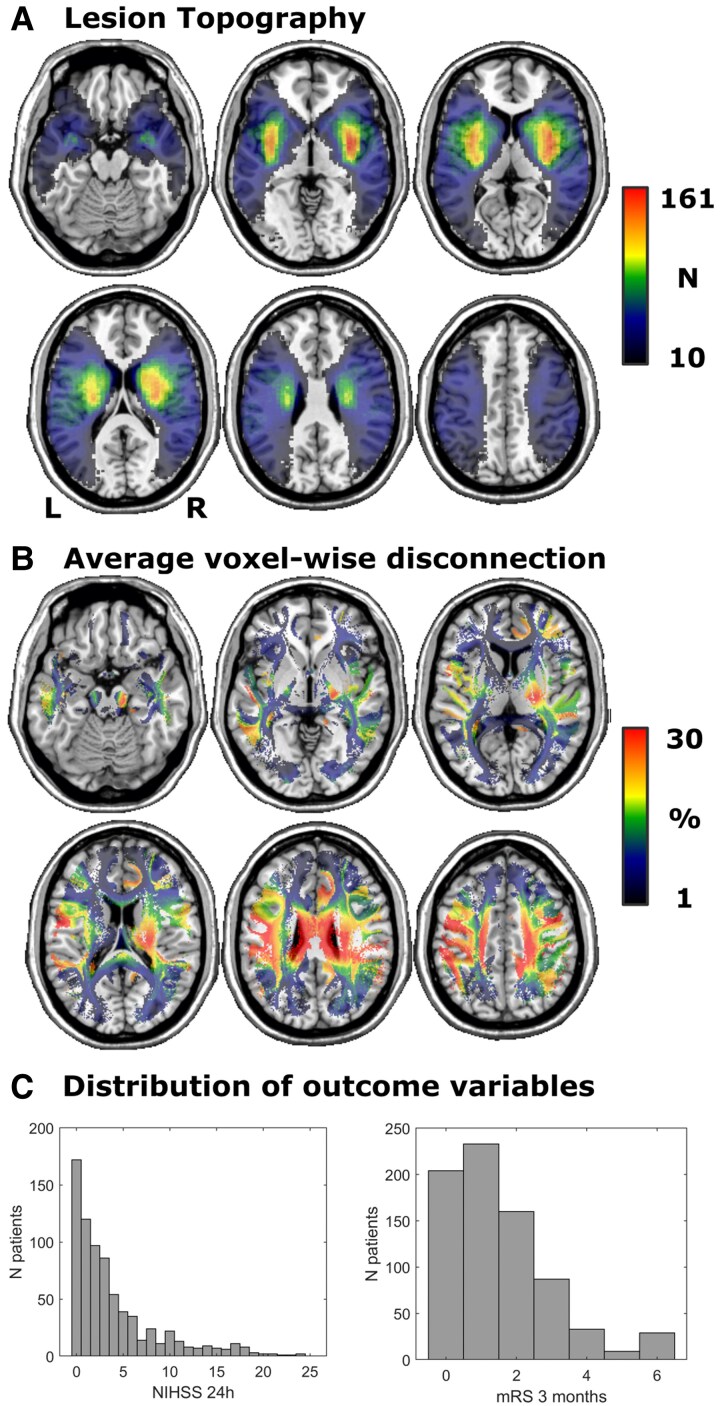
**Descriptives of the main study data.** (**A**) Overlap topography of normalized, binary lesion maps indicating the number of patients with a lesion in each voxel. (**B**) Average voxel-wise disconnection rates in the disconnection maps. (**C**) Histograms of NIHSS at 24 h and mRS at 3 months. Colourblind-friendly versions of panels **A** + **B** are available in Supplementary Fig. 5. NIHSS, National Institutes of Health Stroke Scale; mRS, modified Rankin Scale.

The study enrolled 755 patients, whose demographic and clinical data are shown in Table 1. The distributions of stroke severity (NIHSS at 24 h) and functional outcome (mRS at 3 months), as well as lesion topographies, are shown in Fig. 2. The proportion of patients with favourable functional outcome (mRS < 2) was 57.9%, making the chance classification level 57.9%. Stroke severity and functional outcome were moderately correlated (Pearson’s *r* = 0.56, *P* < 0.0001; Kendall’s *τ* = 0.43, *P* < 0.0001).

**Table 1:** Demographic and clinical data

	All participants, *N* = 755
Age, years mean (SD; range)	66.9 (15.3; 16–95)
Sex(F/M); % male	312/443; 58.7%
NIHSS on admission, median (IQR)	6 (3; 12)
NIHSS 24 h, median (IQR)	2 (1; 5)
mRS 3 months, median(IQR)	1 (0; 2)
mRS 3 months ≥ 2, %	42.1
Lesion size in cm³, median (IQR)	17.4 (4.5; 48.4)
Stroke laterization left/right, %	53.0; 47.0
Acute therapy IVT/MT/both/none, %	28.7/29.7/22.7/18.8
History of TIA, %	4.7
Hypertension, %	66.9
Diabetes, %	15.4
Smoking, %	27.9
Hyperlipidaemia, %	69.9
Atrial fibrillation, %	25.4
Coronary heart disease, %	16.8

Risk factors included missing values (not more than 5.0% of the total sample) that were omitted in the computation of characteristics. IQR, interquartile range; IVT, intravenous thrombolysis; MT, mechanical thrombectomy; SD, standard deviation; NIHSS, National Institutes of Health Stroke Scale; mRS, modified Rankin Scale; TIA, transient ischaemic attack.

### Main results: prediction of stroke outcome

**Figure 3:**
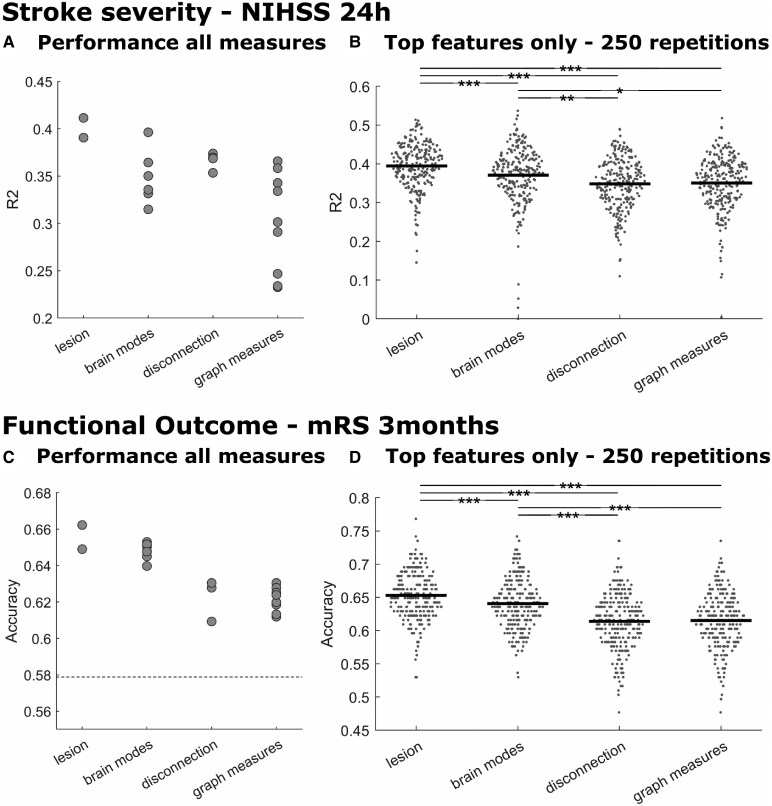
**Prediction performance of spatial and connectomic measures.** Out-of-sample prediction results for (**A** + **B**) acute stroke severity and (**C** + **D**) functional outcome. Panels **A** and **C** show the prediction performance of each data representation, resulting from five repetitions across 5-folds for each dataset. Each point stands for the overall performance of one data representation for each measure category. The dashed line indicates a chance level accuracy of 57.9%. Panels **B** and **D** show the prediction performance for the nominally best predicting data representation in each category in 250 repetitions. Each dot indicates the prediction performance within a fold of 151 patients; bars show the mean. Asterisks indicate statistically significant differences in paired *t*-tests after Bonferroni correction for multiple comparisons. *P < 0.05; **P < 0.01; ***P < 0.001. NIHSS, National Institutes of Health Stroke Scale; mRS, modified Rankin Scale.

A summary of the results is shown in Fig. 3 and Table 2. Detailed information is provided in the supplementary, including additional information on each feature set.

**Table 2:** Summary of model performances across data representations

Type	Measure	Prediction stroke severity, NIHSS 24 h; *R*²	Prediction functional outcome, mRS ≥ 2; % accuracy
Lesion	Spatial lesion features	**0.411**	**66.2**
	Lesion size	0.391	64.9
Brain mode	Unicity	0.350	64.5
	Equivalence	0.332	65.0
	Association	0.315	64.0
	Inhibition	0.336	65.2
	Summation	**0.396**	**65.3**
	All brain modes	0.364	64.8
Structural disconnection	Tract-wise disconnection	**0.374**	60.9
	Disconnection maps	0.353	62.8
	Interregional disconnectome	0.369	**63.1**
	Binary interregional connectome	0.370	62.8
Graph	Δdegree	0.358	62.8
	Δclustering coefficient	0.334	62.5
	Δrich club coefficient	0.247	61.2
	Δdistance	0.301	62.0
	Binary Δdistance	0.343	**63.0**
	Global efficiency	0.233	61.9
	Characteristic path length	0.234	61.3
	Local efficiency	**0.366**	62.4
	Betweenness centrality	0.291	62.4

Overview of all measures used as predictors. Best-performing models in each category are highlighted in bold. For more details, see the supplementary.

The nominally best-performing models both for the regression of acute stroke severity (NIHSS 24 h, *R*² = 0.411) and the classification of favourable versus poor functional outcome (mRS, accuracy = 66.2%) used ‘spatial lesion features’, i.e. voxel-wise lesion location. For brain modes, ‘summation’, i.e. the gradual representation of region pair-wise lesion loads, was the best predictor for both variables. For structural disconnection, the best model to predict the NIHSS 24 h was ‘tract-wise disconnection’, and to predict the mRS, the ‘interregional disconnectome’. For graph measures, ‘local efficiency’, i.e. a measure of how well the neighbours of a region are connected, was best in predicting the NIHSS 24 h, and ‘binary Δdistance’, i.e. a measure of the existence of changes in the shortest structural path length between regions, in classifying mRS. Prediction performances partially varied highly within categories, for example, between *R*² = 0.23 and *R*² = 0.37 for the regression of NIHSS 24 h from graph measures. This variation was influenced by a few imaging markers that resulted in particularly poor performance. Even though the performance of the best-performing models of each category was relatively close (maximal Δ = 4.5% of explained variance/3.2% prediction accuracy), there was no clearly superior data representation of the lesion information.

To support this descriptive evaluation by statistical tests, we repeated the modelling procedure iteratively with the best-performing data representation of each category. One-way analysis of variance suggested a varying performance of feature sets in the prediction of stroke severity (*F*(3996) = 25.2; *P* < 0.0001) and functional outcome (*F*(3996) = 63.4; *P* < 0.0001). Corrected statistical comparisons between groups found the same pattern of results as the previous analysis for the prediction of NIHSS 24 h and classification of mRS 3 months: ‘spatial lesion features’ significantly outperformed all other feature representations and achieved *R*² = 0.395 ± 0.059 in the regression of acute stroke severity and an accuracy of 65.62% ± 3.45 (positive predictive value = 0.72 ± 0.13; negative predictive value = 0.64 ± 0.04) in the classification of functional outcome. Brain modes came second, followed by disconnection and graph measures, which did not differ. Detailed statistics are provided in Supplementary Tables 2–4.

### Control analysis: prediction of specific cognitive deficits

**Figure 4:**
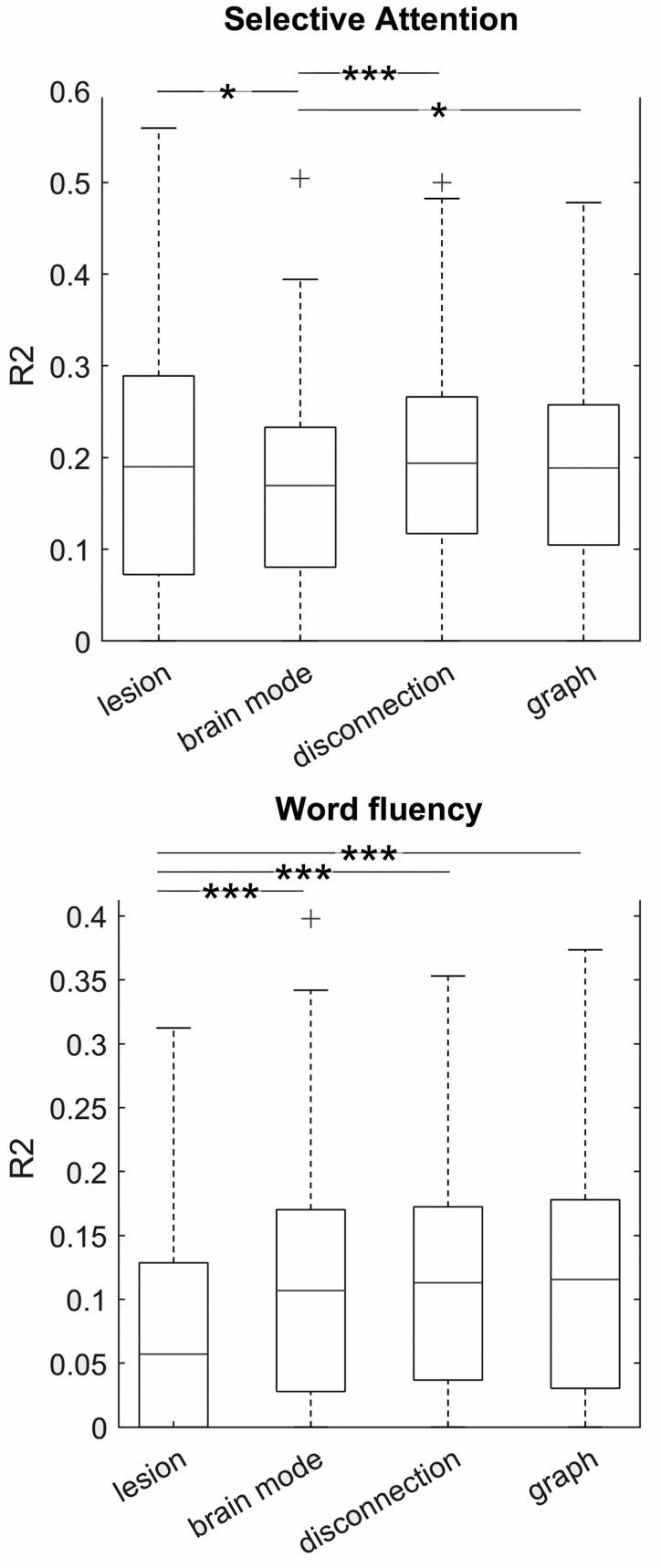
**Results of control analyses.** Prediction performance in the additional sample of ∼180 patients for selective attention and phonemic word fluency. Due to skewness, data were analysed with non-parametric tests, and results are visualized with boxplots showing the median and interquartile range. Asterisks indicate statistically significant differences in paired *t*-tests after Bonferroni correction for multiple comparisons. *P < 0.05; ***P < 0.001.

The prediction of specific cognitive deficits—selective attention and phonemic word fluency—yielded varying results (Fig. 4). For selective attention, the best data representations of each category performed equally well, except for brain modes, which were slightly inferior to other data representations. For word fluency, brain modes, disconnection and graph measures derived from disconnection performed equally well, while lesion features performed worse. In summary, the pattern of results from the main analysis was not replicated in the prediction of specific cognitive deficits. See the supplementary including Supplementary Table 5 for more details.

### Sensitivity analyses

The baseline model with clinical and demographic predictors only (age, sex, NIHSS 24 h, stroke laterality L/R and acute therapy yes/no), but without any imaging variables, reached an accuracy of 70.04% ± 3.71 (positive predictive value = 0.79 ± 0.08; negative predictive value = 0.68 ± 0.04). Thereby, the baseline model outperformed the best model using imaging markers based on spatial lesion features (*t*(249) = 15.95; *P* < 0.0001). This performance, however, relied almost exclusively on the inclusion of neurological baseline deficits as measured by the NIHSS 24 h as a predictor. Without considering the NIHSS 24 h, the baseline model accuracy dropped to 57.15% ± 3.78 (positive predictive value = 0.04 ± 0.11; negative predictive value = 0.58 ± 0.04), meaning that classification performance dropped to the chance level.

Baseline models with low-dimensional lesion parameters only—lesion size and lesion lateralization—were significantly inferior to models with high-dimensional lesion data, though numeric differences between the two were only small. For more details, see Supplementary Fig. 4.

Exclusion of patients with an mRS = 6 did only marginally affect the classification of poor outcome. Detailed results are reported in Supplementary Table 6.

The criterion to define existing connections in the ‘binary interregional connectome’ appeared to not negatively affect the results of our study. The predictive value of the ‘binary interregional connectome’ was not inferior to our original criterion *N*_Streamlines_ = 1 compared to other investigated criteria. For details, see Supplementary Table 7.

## Discussion

We evaluated the predictive value of 21 different stroke imaging feature sets that represent lesion-induced brain perturbation spatially or on low- to high-level network levels. Spatial lesion features, i.e. the simple topography of the lesion, were the best predictors both for acute stroke severity and functional outcome at 3 months. Indirectly estimated connectomic stroke imaging markers provided no benefit in the prediction of global stroke outcome.

The perspective on brain lesions as a perturbation of a complex network is currently highly popular and not only used to understand the genesis of functional deficits after neuronal damage but also considered to potentially provide a clinically useful biomarker.^8-11,27,28^ However, the predictive value of connectomic stroke outcome markers that are indirectly estimated from the structural lesion varies.^10,21,44^ Our study did not find any benefit of connectivity measures for global stroke outcome prognosis: stroke severity and functional outcome are well-represented by spatial lesion imaging features. Clinical studies on global stroke outcome may not require accounting for disconnection, and even the simple size of a brain lesion may suffice as a meaningful variable in statistical models.^40^ However, our control analyses suggested that this finding is not to be generalized to granular post-stroke deficits. While prediction with spatial lesion features and disconnection measures performed equally for selective attention deficits, spatial lesion features were even inferior in the prediction of word fluency deficits.

Why did connectomic measures fail to provide any benefit in the prediction of global stroke outcome? First, all connectomic measures in the current study were derived from the structural imaging of the lesion area. Even with the inclusion of reference data—the healthy streamline atlas—no new information was added; instead, the information contained within the lesion topography was just restructured. However, the many studies that successfully used indirectly estimated connectomic markers indicate that such restructuring can indeed yield more meaningful data.^8-11,13^ Such a benefit of connectomic imaging markers could be deficit-specific. The performance of spatial lesion features against functional connectomic features was previously found to vary between low- and high-level cognitive functions,^45^ and findings on the comparison of structural disconnection and spatial lesion features vary.^10,11,21,44^ In the end, disconnection might be a useful imaging marker especially for those deficits that are considered disconnection syndromes or higher-level cognitive functions that rely on large-scale brain networks, but less so for other deficits or the global impact of stroke. Such interpretation is supported by our additional analyses on specific deficits. Further, the global impact of stroke summarizes the impact of many granular deficits. Therefore, the global impact of stroke might not be attributable to the perturbation of a confined set of connections or local networks, but to the damage resulting from highly differing lesion patterns. Previous works found that global stroke outcome is to some degree associated with specific lesion locations,^46,47^ but fine-grained network measures may still have difficulty in representing this multiplicity of causes, and simpler features, such as spatial lesion features, may better handle these difficulties. This also means that a representation of lesion imaging data with complex connectomic measures might be warranted only when specific and granular post-stroke outcomes are predicted.

Although brain modes were postulated to underlie brain–behaviour associations,^30^ we did not find that their representation improved the prediction of stroke outcomes over simple lesion location. While this could mean that the concept of brain modes in its current form does not adequately represent the global functional impact of stroke, it might also be the case that the non-linear models with lesion imaging features implicitly already represented brain modes; hence, their explicit inclusion did not improve predictions. For both predicted variables, the ‘summation’ brain mode had the nominally best prediction performance, suggesting that this brain mode might be the most representative of general brain function. Further studies are needed to better understand if and how brain modes should be assumed to represent general functional brain architecture or, instead, only specific cases such as spatial attention in the Sprague effect.^31^

Even though our results could not confirm the superiority of advanced imaging markers over spatial topographic lesion features for global stroke outcome prognosis, we are optimistic that further improvements in prognosis are possible. Connectomic measures could profit more from future improvements and could catch up or even reverse the situation. First, our study may have evaluated a large set of connectivity measures, but still only included a subset of all existing measures. Other representations of a stroke lesion exist, of which some vary the featurization of structural disconnection,^34,35^ while others include direct or indirect measures of functional connectivity.^32,33,36^ Directly acquired connectomic data might provide better predictors, but at the price of dedicated and complex imaging sequences and post-processing, and hence lower clinical applicability. Second, it remains an open question if any imaging markers provide complementary information, as previously shown in aphasia.^13^ Third, the processing of high-dimensional imaging data and the selection and tuning of prediction algorithms offer an enormous number of methodological degrees of freedom, which, in the current study, included the focus on structural connectivity measures, the choice of a specific connectome reference atlas, data processing with a specific toolbox and the utilization of only one prediction algorithm. Especially with large consortia datasets that allow for the tuning of various hyperparameters and the application of deep learning, predictions might be further improved. Fourth, the informative value of imaging markers might be limited in the context of stroke outcome prognosis simply because other clinical or demographic variables likewise play an important role, such as acute baseline deficits, brain resilience or brain reserve,^48,49^ comorbidity and rehabilitation. While our study aimed to compare the predictive value of different types of lesion imaging markers—and not to optimize overall prediction performance—any clinically applied algorithm likely has to account for at least some of these variables to provide a significant clinical value. In additional analyses, the current study found that especially neurological baseline stroke deficits, i.e. NIHSS 24 h, may provide a strong predictor for stroke outcome at 3 months.

## Supplementary Material

fcaf268_Supplementary_Data

## Data Availability

Analysis scripts are available at Mendeley Data DOI: 10.17632/jww4w5jnx9.1. Original clinical data are not publicly available, but qualified researchers may contact author R.M.U. (roza.umarova@insel.ch) to request access to anonymized data. Proposals need to be approved by the local ethics committee.

## References

[1] Lansberg MG, Straka M, Kemp S, et al MRI profile and response to endovascular reperfusion after stroke (DEFUSE 2): A prospective cohort study. Lancet Neurol. 2012;11(10):860–867. 22954705 10.1016/S1474-4422(12)70203-X PMC4074206

[2] El-Koussy M, Schroth G, Brekenfeld C, Arnold M. Imaging of acute ischemic stroke. Eur Neurol. 2014;72(5–6):309–316. 25323674 10.1159/000362719

[3] Price CJ, Seghier ML, Leff AP. Predicting language outcome and recovery after stroke: The PLORAS system. Nat Rev Neurol. 2010;6(4):202–210. 20212513 10.1038/nrneurol.2010.15 PMC3556582

[4] Xu T, Jäger HR, Husain M, Rees G, Nachev P. High-dimensional therapeutic inference in the focally damaged human brain. Brain. 2018;141(1):48–54. 29149245 10.1093/brain/awx288 PMC5837627

[5] Rondina JM, Hyun PC, Ward NS. Brain regions important for recovery after severe post-stroke upper limb paresis. J Neurol Neurosurg Psychiatry. 2017;88(9):737–743. 28642286 10.1136/jnnp-2016-315030 PMC5561379

[6] Weaver NA, Kuijf HJ, Aben HP, et al Strategic infarct locations for post-stroke cognitive impairment: A pooled analysis of individual patient data from 12 acute ischaemic stroke cohorts. Lancet Neurol. 2021;20(6):448–459. 33901427 10.1016/S1474-4422(21)00060-0

[7] Hope TMH, Seghier ML, Prejawa S, Leff AP, Price CJ. Distinguishing the effect of lesion load from tract disconnection in the arcuate and uncinate fasciculi. Neuroimage. 2016;125:1169–1173. 26388553 10.1016/j.neuroimage.2015.09.025 PMC4692449

[8] Kuceyeski A, Navi BB, Kamel H, et al Structural connectome disruption at baseline predicts 6-months post-stroke outcome. Hum Brain Mapp. 2016;2601:2587–2601. 10.1002/hbm.23198 PMC4905801 27016287

[9] Griffis JC, Metcalf NV, Corbetta M, Shulman GL. Structural disconnections explain brain network dysfunction after stroke. Cell Rep. 2019;28(10):2527–2540.e9. 31484066 10.1016/j.celrep.2019.07.100 PMC7032047

[10] Salvalaggio A, De Filippo De Grazia M, Zorzi M, Thiebaut de Schotten M, Corbetta M. Post-stroke deficit prediction from lesion and indirect structural and functional disconnection. Brain. 2020;143(7):2173–2188. 32572442 10.1093/brain/awaa156 PMC7363494

[11] Talozzi L, Forkel SJ, Pacella V, et al Latent disconnectome prediction of long-term cognitive-behavioural symptoms in stroke. Brain. 2023;146(5):1963–1978. 36928757 10.1093/brain/awad013 PMC10151183

[12] Kuceyeski A, Maruta J, Relkin N, Raj A. The network modification (NeMo) tool: Elucidating the effect of white matter integrity changes on cortical and subcortical structural connectivity. Brain Connect. 2013;3(5):451–463. 23855491 10.1089/brain.2013.0147 PMC3796322

[13] Pustina D, Coslett HB, Ungar L, et al Enhanced estimations of post-stroke aphasia severity using stacked multimodal predictions. Hum Brain Mapp. 2017;38(11):5603–5615. 28782862 10.1002/hbm.23752 PMC5765865

[14] Foulon C, Cerliani L, Kinkingnéhun S, et al Advanced lesion symptom mapping analyses and implementation as BCBtoolkit. Gigascience. 2018;7(3):1–17. 10.1093/gigascience/giy004 PMC5863218 29432527

[15] Griffis JC, Metcalf NV, Corbetta M, Shulman GL. Lesion Quantification Toolkit: A MATLAB software tool for estimating grey matter damage and white matter disconnections in patients with focal brain lesions. NeuroImage Clin. 2021;30:102639. 33813262 10.1016/j.nicl.2021.102639 PMC8053805

[16] Sperber C, Griffis J, Kasties V. Indirect structural disconnection-symptom mapping. Brain Struct Funct. 2022;227(9):3129–3144. 36048282 10.1007/s00429-022-02559-x

[17] Wawrzyniak M, Stockert A, Klingbeil J, Saur D. Voxelwise structural disconnection mapping: Methodological validation and recommendations. NeuroImage Clin. 2022;35:103132. 36002968 10.1016/j.nicl.2022.103132 PMC9421530

[18] Smits AR, van Zandvoort MJE, Ramsey NF, de Haan EHF, Raemaekers M. Reliability and validity of DTI-based indirect disconnection measures. NeuroImage Clin. 2023;39(10):103470. 37459698 10.1016/j.nicl.2023.103470 PMC10368919

[19] Rivier C, Preti MG, Nicolo P, Van De Ville D, Guggisberg AG, Pirondini E. Prediction of post-stroke motor recovery benefits from measures of sub-acute widespread network damages. Brain Commun. 2023;5(2):1–13. 10.1093/braincomms/fcad055 PMC10016810 36938525

[20] Bonilha L, Gleichgerrcht E, Nesland T, Rorden C, Fridriksson J. Success of anomia treatment in aphasia is associated with preserved architecture of global and left temporal lobe structural networks. Neurorehabil Neural Repair. 2016;30(3):266–279. 26150147 10.1177/1545968315593808 PMC4703576

[21] Griffis JC, Metcalf NV, Corbetta M, Shulman GL. Damage to the shortest structural paths between brain regions is associated with disruptions of resting-state functional connectivity after stroke. Neuroimage. 2020;210:116589. 32007498 10.1016/j.neuroimage.2020.116589 PMC7061444

[22] Zevgolatakou E, Thye M, Mirman D. Behavioural and neural structure of fluent speech production deficits in aphasia. Brain Commun. 2022;5(1):1–16. 10.1093/braincomms/fcac327 PMC9798301 36601623

[23] Rubinov M, Sporns O. Complex network measures of brain connectivity: Uses and interpretations. Neuroimage. 2010;52(3):1059–1069. 19819337 10.1016/j.neuroimage.2009.10.003

[24] Bullmore E, Sporns O. Complex brain networks: Graph theoretical analysis of structural and functional systems. Nat Rev Neurosci. 2009;10(3):186–198. 19190637 10.1038/nrn2575

[25] Bernhardt BC, Bonilha L, Gross DW. Network analysis for a network disorder: The emerging role of graph theory in the study of epilepsy. Epilepsy Behav. 2015;50:162–170. 26159729 10.1016/j.yebeh.2015.06.005

[26] Caeyenberghs K, Verhelst H, Clemente A, Wilson PH. Mapping the functional connectome in traumatic brain injury: What can graph metrics tell us? Neuroimage. 2017;160:113–123. 27919750 10.1016/j.neuroimage.2016.12.003

[27] Fornito A, Zalesky A, Breakspear M. Graph analysis of the human connectome: Promise, progress, and pitfalls. Neuroimage. 2013;80:426–444. 23643999 10.1016/j.neuroimage.2013.04.087

[28] Vogel JW, Corriveau-Lecavalier N, Franzmeier N, et al Connectome-based modelling of neurodegenerative diseases: Towards precision medicine and mechanistic insight. Nat Rev Neurosci. 2023;24(10):620–639. 37620599 10.1038/s41583-023-00731-8

[29] Godefroy O, Duhamel A, Leclerc X, Saint Michel T, Hénon H, Leys D. Brain-behaviour relationships. Some models and related statistical procedures for the study of brain-damaged patients. Brain. 1998;121(Pt 8):1545–1556. 9712015 10.1093/brain/121.8.1545

[30] Toba MN, Godefroy O, Rushmore RJ, et al Revisiting ‘brain modes’ in a new computational era: Approaches for the characterization of brain-behavioural associations. Brain. 2020;143(4):1088–1098. 31764975 10.1093/brain/awz343 PMC7174035

[31] Valero-Cabré A, Toba MN, Hilgetag CC, Rushmore RJ. Perturbation-driven paradoxical facilitation of visuo-spatial function: Revisiting the ‘Sprague effect’. Cortex. 2020;122:10–39. 30905382 10.1016/j.cortex.2019.01.031

[32] Boes AD, Prasad S, Liu H, et al Network localization of neurological symptoms from focal brain lesions. Brain. 2015;138(Pt 10):3061–3075. 26264514 10.1093/brain/awv228 PMC4671478

[33] Allegra M, Favaretto C, Metcalf N, Corbetta M, Brovelli A. Stroke-related alterations in inter-areal communication. NeuroImage Clin. 2021;32:102812. 34544032 10.1016/j.nicl.2021.102812 PMC8453222

[34] Mrah S, Descoteaux M, Wager M, et al Network-level prediction of set-shifting deterioration after lower-grade glioma resection. J Neurosurg. 2022;137:1–9. 35245898 10.3171/2022.1.JNS212257

[35] Wilmskoetter J, He X, Caciagli L, et al Language recovery after brain injury: A structural network control theory study. J Neurosci. 2022;42(4):657–669. 34872927 10.1523/JNEUROSCI.1096-21.2021 PMC8805614

[36] Idesis S, Allegra M, Vohryzek J, et al A low dimensional embedding of brain dynamics enhances diagnostic accuracy and behavioral prediction in stroke. Sci Rep. 2023;13(1):15698. 37735201 10.1038/s41598-023-42533-z PMC10514061

[37] Umarova RM, Sperber C, Kaller CP, et al Cognitive reserve impacts on disability and cognitive deficits in acute stroke. J Neurol. 2019;266(10):2495–2504. 31254064 10.1007/s00415-019-09442-6

[38] Yeh FC, Panesar S, Fernandes D, et al Population-averaged atlas of the macroscale human structural connectome and its network topology. Neuroimage. 2018;178:57–68. 29758339 10.1016/j.neuroimage.2018.05.027 PMC6921501

[39] Fan L, Li H, Zhuo J, et al The Human Brainnetome Atlas: A new brain atlas based on connectional architecture. Cereb Cortex. 2016;26(8):3508–3526. 27230218 10.1093/cercor/bhw157 PMC4961028

[40] Sperber C, Gallucci L, Mirman D, Arnold M, Umarova RM. Stroke lesion size—Still a useful biomarker for stroke severity and outcome in times of high-dimensional models. NeuroImage Clin. 2023;40:103511. 37741168 10.1016/j.nicl.2023.103511 PMC10520672

[41] Ganesh A, Luengo-Fernandez R, Wharton RM, Rothwell PM. Ordinal vs dichotomous analyses of modified Rankin scale, 5-year outcome, and cost of stroke. Neurology. 2018;91(21):e1951–e1960. 30341155 10.1212/WNL.0000000000006554 PMC6260198

[42] Röhrig L, Sperber C, Bonilha L, Rorden C, Karnath HO. Right hemispheric white matter hyperintensities improve the prediction of spatial neglect severity in acute stroke. NeuroImage Clin. 2022;36:103265. 36451368 10.1016/j.nicl.2022.103265 PMC9723300

[43] Gallucci L, Sperber C, Guggisberg AG, et al Post-stroke cognitive impairment remains highly prevalent and disabling despite state-of-the-art stroke treatment. Int J Stroke. 2024;19(8):888–897. 38425239 10.1177/17474930241238637

[44] Hope TMH, Leff AP, Price CJ. Predicting language outcomes after stroke: Is structural disconnection a useful predictor? NeuroImage Clin. 2018;19:22–29. 30034998 10.1016/j.nicl.2018.03.037 PMC6051761

[45] Siegel JS, Ramsey LE, Snyder AZ, et al Disruptions of network connectivity predict impairment in multiple behavioral domains after stroke. Proc Natl Acad Sci U S A. 2016;113(30):E4367–E4376. 27402738 10.1073/pnas.1521083113 PMC4968743

[46] Cheng B, Forkert ND, Zavaglia M, et al Influence of stroke infarct location on functional outcome measured by the modified Rankin scale. Stroke. 2014;45(6):1695–1702. 24781084 10.1161/STROKEAHA.114.005152

[47] Ernst M, Boers AMM, Forkert ND, et al Impact of ischemic lesion location on the MRS score in patients with ischemic stroke: A voxel-based approach. AJNR Am J Neuroradiol. 2018;39(11):1989–1994. 30287456 10.3174/ajnr.A5821 PMC7655370

[48] Umarova RM . Adapting the concepts of brain and cognitive reserve to post-stroke cognitive deficits: Implications for understanding neglect. Cortex. 2017;97:327–338. 28049565 10.1016/j.cortex.2016.12.006

[49] Liew SL, Schweighofer N, Cole JH, et al Association of brain age, lesion volume, and functional outcome in patients with stroke. Neurology. 2023;100(20):E2103–E2113. 37015818 10.1212/WNL.0000000000207219 PMC10186236

