# Supplementary material for: Connectomic stroke lesion measures provide no benefit over basic spatial lesion features in the prognosis of global stroke outcome measures
Source: Brain Commun. 2025 Jul 28;7(4):fcaf268. doi: 10.1093/braincomms/fcaf268 (PMC12301883; doi:10.1093/braincomms/fcaf268)
Supplement: fcaf268_Supplementary_Data [file fcaf268_supplementary_data.pdf]

Supplementary

Contents

Supplementary Figure 1 2
Optimisation of binary cut-off for interregional disconnection 3
Supplementary Figure 2 4
Details on the computation of graph measures 4
Optimisation of binary cut-off for region-wise lesion load 5
Supplementary Figure 3 6
Details on hyperparameter optimisation 6
Post-hoc analysis – a simple prediction model with lesion volume and laterality 7
Supplementary Figure 4 8
Control analyses: prediction of specific cognitive deficits 8
Supplementary Figure 5 11
Supplementary Table 1: Detailed total overview of lesion and connectomic measures 12
Supplementary Table 2: Detailed results of the repeated modelling procedure for acute stroke severity 14
Supplementary Table 3: Detailed results of the repeated modelling procedure for prediction of functional outcome 15
Supplementary Table 4: Detailed statistical comparisons of the repeated modelling procedure 16
Supplementary Table 5: Detailed results of the additional prediction analyses of specific cognitive deficits 17
Supplementary Table 6: Results of the control analysis when excluding patients with an mRS=6 19
Supplementary Table 7: Results of the control analysis with other criteria to define an existing connection in the binary interregional connectome 20

Supplementary Figure 1

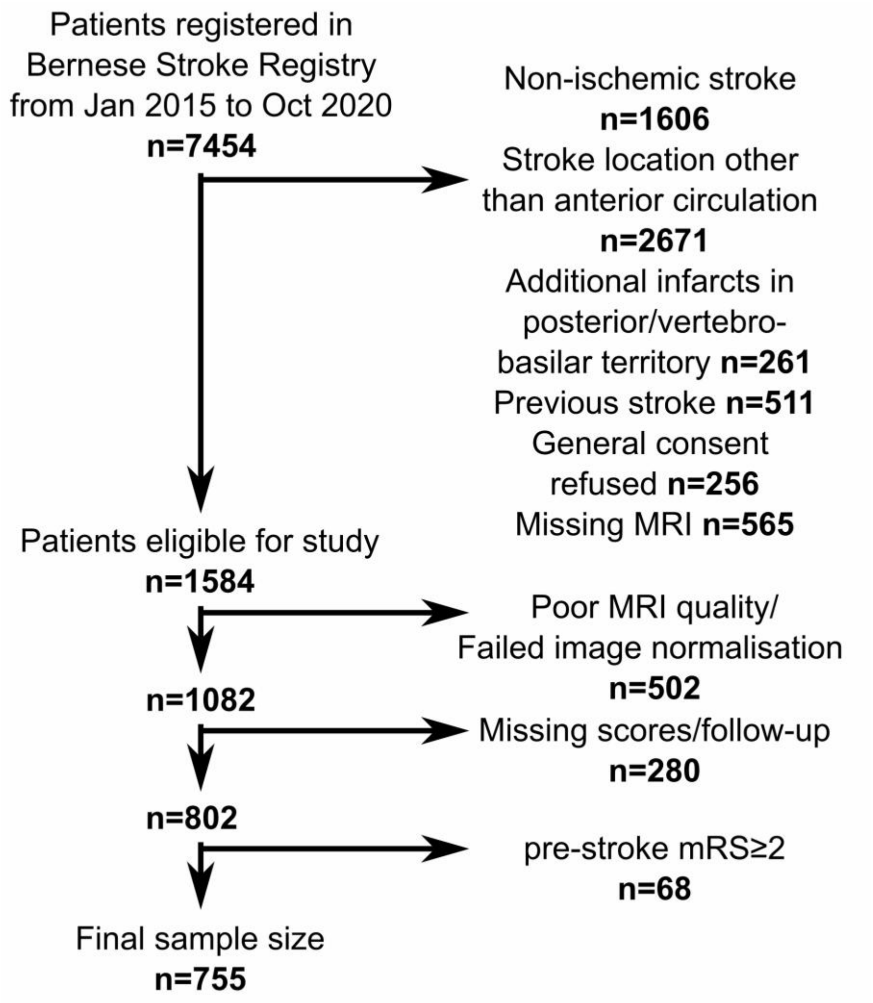

Supplementary Figure 1: Patient recruitment flowchart

Optimisation of binary cut-off for interregional disconnection

Many graph measures are only defined for binary connectivity matrices. However, the interregional disconnection matrices generated by the Lesion Quantification Toolkit contained proportional values (i.e. between 0 and 1) of the number of disconnected streamlines. We copied the strategy of a previous study that successfully created graph imaging markers from interregional disconnection matrices (Griffis et al., 2020) and binarised the matrices at a certain cut-off. However, contrary to this previous study, we did not select any arbitrary measures but selected a cut-off based on the association with our outcome measure (stroke severity, NIHSS 24 h). We tested cut-offs from 5% to 95% in steps of 5%. For each interregional pair with any disconnection in at least 25 out of the 755 patients, we compared via a two-sample t-test the NIHSS 24h between all patients with a disconnection given the cut-off against patients with an intact connection. We searched for a cut-off for which the average t-statistic in the top 50 connections was maximal. In other words, we aimed to find a cut-off for which the binarised disconnection status was most strongly associated with stroke severity. The results are shown in the supplementary Figure below. The t-statistics were maximised with a cut-off of 90%, i.e. when interregional connections with at least 90% of disconnected streamlines were considered disconnected. Note that this procedure, for simplicity of the design, was only done once within the sample, potentially, but unlikely, creating a slightly over-optimistic predictor of stroke severity.

Supplementary Figure 2

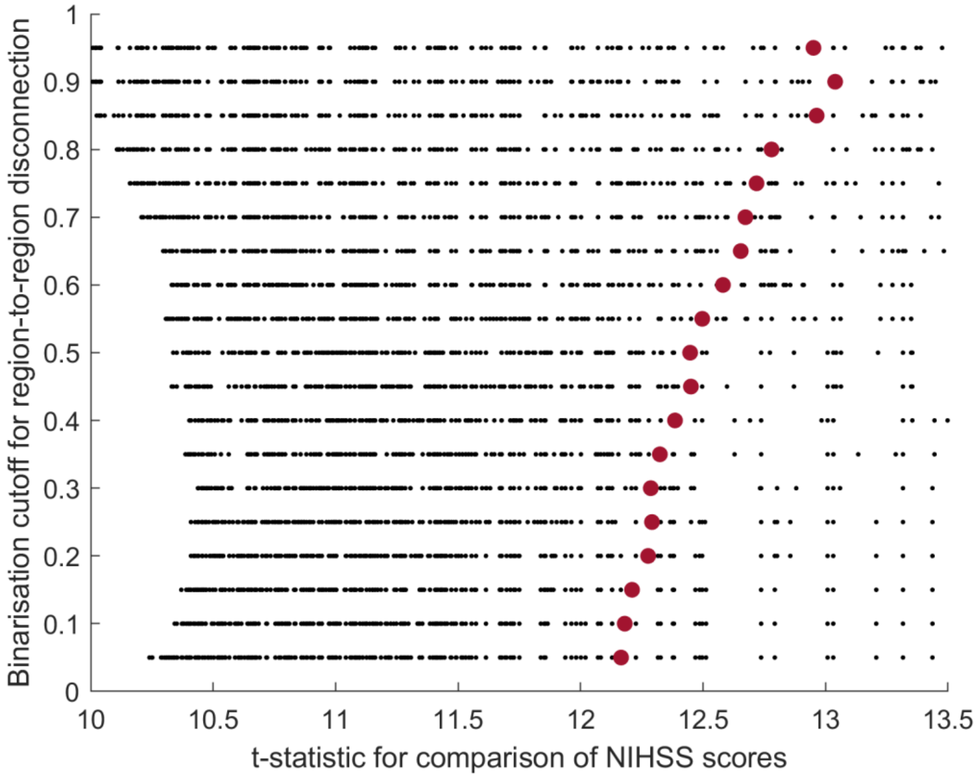

Supplementary Figure 2: t-statistics for comparing NIHSS 24h between intact and disrupted connections across different cut-offs to define disconnection from the proportion of disrupted streamlines. For each cut-off, the 200 largest t-statistics are shown; the red dot indicates the mean of the 50 largest t-statistics, which was the target criterion in the optimisation procedure.

Details on the computation of graph measures

The computation of graph measures required some additional steps to generate potentially meaningful markers from a lesioned binary interregional connectome. For several graph measures, we aimed to represent the network's perturbation caused by the lesion-induced disconnection and not the overall status of the network. To do so, we computed the graph measures both for the healthy connectome and each patient's perturbed connectome. The healthy connectome was defined by the interregional connectome in the HCP-842 population-averaged streamline atlas. Each pair of regions with at least 1 streamline connecting both regions was considered connected. The patient's spared connectome was created from the proportional interregional disconnection estimated by the Lesion Quantification Toolbox. As a cut-off to define

disconnected regions, we selected a disconnection of at least 90% based on the empirical procedure described above (‘Optimisation of binary cut-off for interregional disconnection’). The final measure of pathology, noted Δ*variable,* was the difference between the graph measure of the healthy connectome and the graph measure of the patient’s spared connectome. For some global measures or measures where disconnection could lead to both positive or negative changes (such as betweeness centrality; Rubinov and Sporns, 2010) we directly used the patient’s graph measures without reference to the healthy connectome.

For the *Δdistance* between nodes (i.e. the path length), we excluded nodes that were not connected at all in the healthy connectome and which, hence, did not allow for the computation of a distance metric. Further, entirely disconnected regions in a patient received an ‘Infinite’ path length. As this would be problematic in any prediction algorithm, we replaced those infinite values with the largest observed path length in the healthy connectome +1. The computation of *characteristic path length* was also performed with these replaced scores to avoid the generation of infinite values. For the *Δrich club coefficient,* we had to provide a hyperparameter k for the number of degrees accounted for by this measure. As no single objectively adequate measure exists, we derived the measure across 8 different degrees [5, 10, 15, 20, 25, 30, 35, 40].

Optimisation of binary cut-off for region-wise lesion load

Most brain modes are defined for binary lesion load, i.e. the representation of a region-wise status as either damaged or intact. Hence, we binarised the lesion load which is the proportion of lesioned voxels in a region. As with the binarisation of interregional disconnection described above, we selected a cut-off based on the association with our outcome measure (stroke severity, NIHSS at 24 h). We tested cut-offs from 5% to 95% in steps of 5%. For each region in the BN-246 parcellation with any lesioned voxel in at least 25 out of the 755 patients, we compared via a two-sample t-test the NIHSS 24h between all patients with a damaged region given the cut-off against patients with an intact region. We aimed to optimise the average magnitude of the 15 largest t-statistics at each cut-off. We chose the number of 15 regions – compared to 50 connections in the equivalent analysis for interregional connections – as the number of regions was smaller than the number of connections. The results are shown in Supplementary Figure 3 below. The t-statistics were

maximised with a cut-off of 60%, i.e. when regions with at least 60% of lesioned voxels were considered damaged.

Supplementary Figure 3

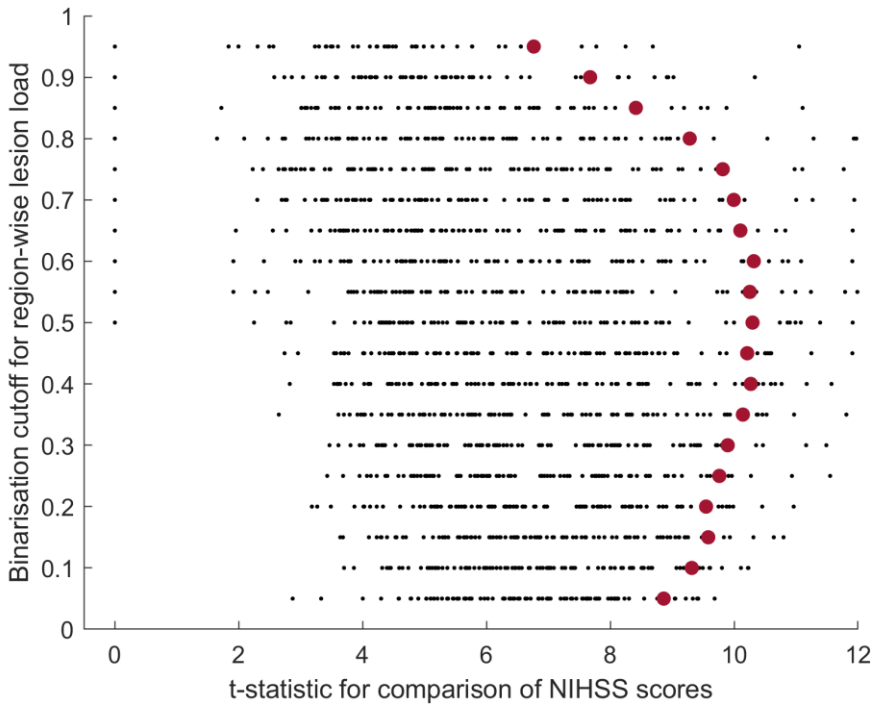

Supplementary Figure 3: t-statistics for comparing NIHSS 24h between intact and lesioned grey matter regions across different cutoffs to define an area to be damaged based on the proportion of lesioned voxels. For each cut-off, the red dot indicates the mean of the 15 largest t-statistics, which was the target criterion in the optimisation procedure.

Details on hyperparameter optimisation

NIHSS 24h was predicted with a *support vector regression* with a radial basis function kernel with the hyperparameters box constraint C, kernel scale γ, and ε. For the classification of functional outcome at 3 months a *support vector machine* with a radial basis function kernel with the hyperparameters box constraint C and kernel scale γ was used. All hyperparameters were optimised by Bayesian optimisation and were chosen within the software's default parameters. The box constraint C (also known as soft margin) was chosen among positive values log-scaled in the range [1e-3,1e3]. Kernel scale γ was chosen among positive values log-scaled in the range [1e-

3,1e3]. ε was chosen among positive values log-scaled in the range [1e-3,1e2]*IQR(Y)/1.349.

Post-hoc analysis – a simple prediction model with lesion volume and laterality

Adequate representation of imaging features with low-dimensional variables could allow for the efficient inclusion of lesion features in statistical models. For example, features such as lesion size or lesion laterality could be included in inferential multiple regression models in clinical studies on stroke interventions or rehabilitation. Therefore, we post hoc added an evaluation of such a low-dimensional model using the predictors lesion volume, lesion laterality, and age to evaluate how well these variables represent the anatomical information derived from structural imaging. We repeated the methodological pipeline reported in the manuscript and subjected the predictors to the nested cross-validation with bootstrapping to estimate the overall model performance and its variability across 250 repetitions (see Supplementary Figure 4). The models achieved a comparatively good prediction of the NIHSS at 24h (R^2=0.373±0.065) and the mRS at 3 months (accuracy=0.646%±0.036). However, this performance was significantly worse than for the model with spatial lesion features both for the NIHSS (t(498)=3.976; p=0.0008) and the mRS (t(498)=3.127; p=0.0019). Hence, the representation of stroke imaging features by these variables appears to be justifiable in simple statistical models, but suboptimal in prediction algorithms.

Supplementary Figure 4

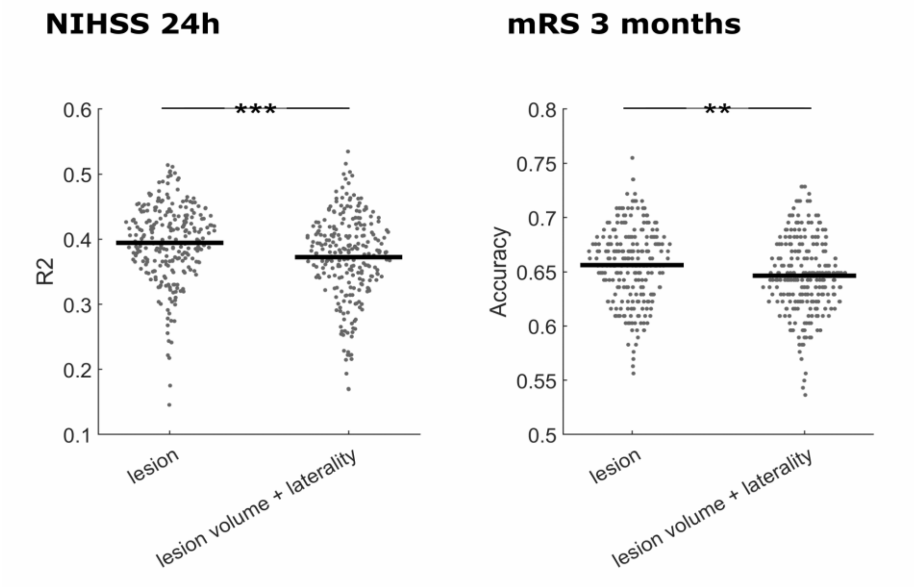

Supplementary Figure 4: Out-of-sample prediction performance of a model with lesion volume, lesion laterality and age as predictors. Each dot indicates the prediction performance within a fold of 151 patients in one out of the 250 repetitions of the modelling procedure; bars show the mean. Asterisks indicate statistically significant differences according to paired t-tests. ** - p<0.01; *** - p<0.001

Control analyses: prediction of specific cognitive deficits

The main analyses focussed on *global* measures of stroke outcome that summarize the main post-stroke neurological deficits such as hemiparesis, aphasia, ataxia etc. To put our main results into the larger context of stroke outcome prediction, we performed an additional control analysis to predict specific cognitive deficits representing *fine-granular* measures. We therefore consulted another sample of stroke patients for which granular cognitive measures were available. The sample was recruited prospectively to track post-stroke cognitive impairment. The project design and detailed recruitment strategy are outlined in the accompanying publication (Gallucci et al., 2024). All patients were admitted to the Stroke Centre in Bern with a first-ever anterior circulation stroke. Patients with previous stroke, aphasia or other neurological conditions restricting neuropsychological assessment, or known pre-stroke cognitive decline were excluded. For information on clinical registration, patient consent, and ethical approval please also see Galluci and colleagues (2024).

Patients were examined in the acute stroke phase within the first 10 days post-stroke. We modelled two specific cognitive deficits: selective attention and phonemic word fluency. *Selective attention* was assessed with the paper-and-pencil Bells Test (Gauthier et al., 1989). Patients were asked to circle bell-shaped black icons placed among distractor items on a vertical sheet of paper (210mm x 297mm). The final score was the total number of omitted targets. Phonemic *verbal fluency* was assessed by asking the patient to generate as many words with a specific starting letter as possible within 60 seconds (alternating for letters G-R, Aschenbrenner et al., 2000).

For inclusion in the current analyses, a normalised, voxel-wise binary lesion map was required. The data processing pipeline to create such mirrored the pipeline reported in the main analyses. At the start of the current analysis, lesion maps were not yet generated for all patients investigated by Gallucci and colleagues. Further, some patients could not be included in the present analysis due to missing cognitive measures. In the the final sample of 182 patients, a measure of selective attention was available for 181 patients and of word fluency for 178 patients.

We derived connectomic lesion measures following the same strategies as in the main analyses. Steps that required cutoffs for the binarisation of a measure used the same cutoffs as in the main analysis to maximise comparability. During piloting runs of the modelling procedures, we found that the computational demands with the smaller samples were much lower, potentially allowing more complex analyses. On the other hand, the validation sample was smaller so prediction performances fluctuated more strongly. Further, the algorithms appeared to struggle to predict deficits from some data representations such as disconnectome maps. Therefore, the first analysis which created a single prediction for each patient from a small number of modelling repetitions provided imprecise estimates of model performance and we decided not to replicate it. Instead, we only replicated the second analysis that used bootstrapping for repeated nested cross-validation. With a high number of repetitions, this procedure should generate precise estimates of prediction performance and variance and allow statistical comparison between data featurisations with high statistical power. To compensate for the high variance in prediction performance, we increased the number of repetitions from 250 to 500. As we did not perform a first analysis to select the top candidate data featurisation in each data category, we included all data representations here instead of just one per category (lesion, brain mode, disconnection and graph). However, to make this massive increase in the

number of prediction models feasible, we omitted some data representations after they performed suboptimal during piloting models for specific cognitive deficits.

The statistical analysis was again limited to the best-performing models in each category. Due to skewness, we now used non-parametric Mann-Whitney tests with Bonferroni correction for multiple comparisons to compare performances.

Results

In this control analysis, 182 patients were included (age 65.8±14.8y, 105m/77f, NIHSS 24h median=1 [IQR=0;3]), in whom neuropsychological assessment was done 2.6±1.9 days post-stroke. For selective attention, the best data representations of each category performed equally well, except for brain modes, which were slightly inferior to other data representations. For word fluency, brain modes, disconnection and graph measures derived from disconnection performed equally well, while lesion features performed worse. In summary, the pattern of results from the main analysis was not replicated in the prediction of specific cognitive deficits. Detailed results across all data representations are reported in Supplementary Table 5.

Supplementary References

Aschenbrenner S, Tucha O, Lange KW, 2000. Regensburger Wortflüssigkeits-Test: RWT. Hogrefe, Verlag für Psychologie.

Gallucci L, Sperber C, Guggisberg AG, et al. Post-stroke cognitive impairment remains highly prevalent and disabling despite state-of-the-art stroke treatment. Int. J. Stroke 2024;00:17474930241238636.

Gauthier L, Dehaut F, Yves J. The Bells Test: A Quantitative and Qualitative Test For Visual Neglect. Int. J. Clin. Neuropsychol. 1989;XI(2):49–54.

Supplementary Figure 5

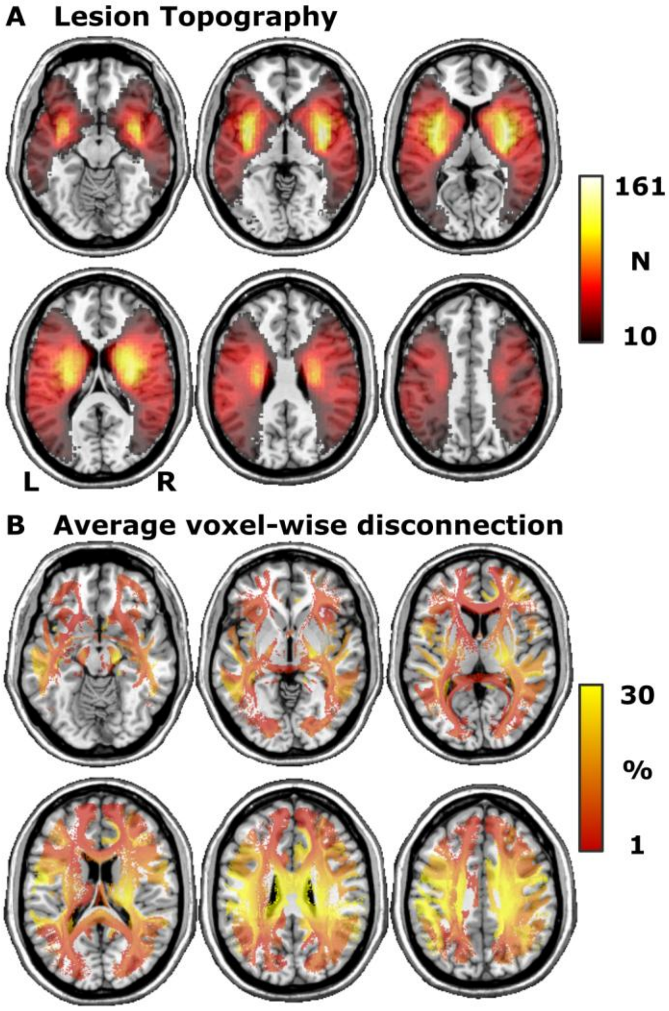

Supplementary Figure 5: Colourblind-friendly version of Figures 2A+B

(A) Overlap topography of normalised, binary lesion maps indicating the number of patients with a lesion in each voxel. **(B)** Average voxel-wise disconnection rates in the disconnection maps.

Supplementary Table 1: Detailed total overview of lesion and connectomic measures

Overview of all measures used as predictors. Best-performing models in each category are highlighted in bold. For large-scale data, feature-reduced sets obtained from scores of a principal component analysis were used. For these, two numbers of features *full features*/*componential features* are reported; the performance of the model is shown in the same way. The total number of features shown is the number of features after removing uninformative ones.

* Features that did carry little to no variance or were redundant were removed. First, features that (almost) always indicated a non-pathological state, i.e. that carried the same value across the entire sample, were removed. Such features are unsuited to contribute information in a cross-validated learning algorithm. We removed features that indicated pathology in less than 10 patients ("n<10"). Second, redundant or uninformative (i.e. on the diagonal of a disconnection matrix) features in symmetric matrices were removed ("redundant"). Third, features that showed no variance ("no variance") were removed, which applied to conceptually meaningless graph measures for a few regions. For brain modes, all 94 regions with lesion to in at least 10 patients were considered, but no further features were excluded, i.e. all brain modes were computed for the same set of brain regions.

** Disconnection maps were down-sampled back into 2x2x2 mm^3 space as the full data were too large to be processed by our hardware.

Type	Measure	Raw Data format	Excluded features*	# of features	Prediction NIHSS R^2	Prediction mRS≥2 %acc.
Lesion	Spatial lesion features	3D image	n<10	97094/12	0.410/ **0.411**	**66.2**/65.3
	Lesion Size (log_{10} transformed)	Single value	-	1	0.391	64.9
Brain Mode	Unicity	Vector (region-wise)	n<10	94	0.350	64.5
	Equivalence	Symmetric matrix	Redundant, n<10	4371	0.332	65.0
	Association	Symmetric matrix	Redundant, n<10	4371	0.315	64.0
	Inhibition	Symmetric matrix	Redundant, n<10	4371	0.336	65.2

	Summation	Symmetric matrix	Redundant, n<10	4371	**0.396**	**65.3**
	All brain modes	Various	Redundant, n<10	17578/12	0.336/ 0.364	64.8/64.0
Structural disconnection	Tract-wise disconnection	Vector (tract-wise)	n<10	52	**0.374**	60.9
	Disconnection maps	3D image	n<10	74017/10 **	0.353/ 0.326	62.7/62.8
	Interregional disconnectome	Symmetric matrix	Redundant, n<10	2973/10	0.369/ 0.341	**63.1**/62.5
	Binary interregional connectome	Symmetric matrix	Redundant, n<10	2973	0.370	62.8
Graph	Δdegree	Vector (region-wise)	n<10	239	0.358	62.8
	Δclustering coefficient	Vector (region-wise)	n<10	221	0.334	62.5
	Δrich club coefficient	Single values at 8 different degrees	-	8	0.247	61.2
	Δdistance	Symmetric matrix	Redundant, n<10	25662	0.301	62.0
	Binary Δdistance	Symmetric matrix	Redundant, n<10	25662	0.343	**63.0**
	Global efficiency	Single value	-	1	0.233	61.9
	Characteristic path length	Single value	-	1	0.234	61.3
	Local efficiency	Vector (region-wise)	No variance	225	**0.366**	62.4
	Betweeness centrality	Vector (region-wise)	No variance	241	0.291	62.4

Supplementary Table 2: Detailed results of the repeated modelling procedure for acute stroke severity

Detailed results for the nominally best predicting data representation in each category as found in the first modelling experiment across 250 repetitions. Mean ± Standard Deviation is shown.

	Stroke severity (NIHSS 24h)	
**Category**	**Measure**	**Prediction R^2**
**Lesion**	Spatial lesion features	0.395 ± 0.059
**Brain Mode**	All combined	0.371 ± 0.074
**Disconnection**	Tract-Wise	0.349 ± 0.066
**Graph**	Local efficiency	0.351 ± 0.071

Supplementary Table 3: Detailed results of the repeated modelling procedure for prediction of functional outcome

Detailed results for the nominally best predicting data representation in each category as found in the first modelling experiment across 250 repetitions. Mean ± Standard Deviation is shown.

	Poor vs. favourable functional Outcome (mRS 3 months)			
**Category**	**Measure**	**Prediction Accuracy %**	**Positive predictive value**	**Negative predictive value**
**Lesion**	Spatial lesion features	65.62 ± 3.45	0.72 ± 0.13	0.64 ± 0.04
**Brain Mode**	Summation	64.41 ± 3.51	0.73 ± 0.11	0.63 ± 0.04
**Disconnection**	Interregional disconnectome	61.91 ± 3.90	0.61 ± 0.17	0.62 ± 0.04
**Graph**	Binary Δdistance	61.97 ± 3.77	0.60 ± 0.18	0.62 ± 0.04

Supplementary Table 4: Detailed statistical comparisons of the repeated modelling procedure

Detailed statistical comparison between the nominally best-predicting data representation in each category as found in the first modelling experiment. Paired t-tests were used to compare performance between models. See supplementary tables 2 and 3 for the mean and standard deviation of all measures.

Feature Set 1	Feature Set 2	Statistic	p (uncorrected)	p (Bonferroni-corrected)
**Stroke Severity – NIHSS 24h**				
**Lesion -** Spatial features	**Brain Mode -** All combined	t(498) = 3.97	0.00008	0.0005
**Lesion -** Spatial features	**Disconnection -** Tract-Wise	t(498) = 8.23	<0.00001	<0.00001
**Lesion -** Spatial features	**Graph -** Local efficiency	t(498) = 7.56	<0.00001	<0.00001
**Brain Mode -** All combined	**Disconnection -** Tract-Wise	t(498) = 3.55	0.0004	0.0025
**Brain Mode -** All combined	**Graph -** Local efficiency	t(498) = 3.13	0.0019	0.0112
**Disconnection -** Tract-Wise	**Graph -** Local efficiency	t(498) = 0.32	0.74	1
**Functional Outcome – mRS 3 months**				
**Lesion -** Spatial features	**Brain Mode -** All combined	t(498) = 3.88	0.0001	0.0007
**Lesion -** Spatial features	**Disconnection -** Tract-Wise	t(498) = 11.27	<0.00001	<0.00001
**Lesion -** Spatial features	**Graph -** Local efficiency	t(498) = 11.30	<0.00001	<0.00001
**Brain Mode -** All combined	**Disconnection -** Tract-Wise	t(498) = 7.54	<0.00001	<0.00001
**Brain Mode -** All combined	**Graph -** Local efficiency	t(498) = 7.50	<0.00001	<0.00001
**Disconnection -** Tract-Wise	**Graph -** Local efficiency	t(498) = 0.17	0.87	1

Supplementary Table 5: Detailed results of the additional prediction analyses of specific cognitive deficits

Detailed results for the prediction of selective attention and word fluency in the additional sample of ~180 patients. Due to skewness, results are reported as median and interquartile interval. Performance measures originate from 500 bootstrapped repetitions of the nested modelling procedure. For large-scale data, feature-reduced sets obtained from scores of a principal component analysis were used. For these, two numbers of features *full features*/*componential features* are reported

Type	Measure	Prediction Selective attention R^2	Prediction Word fluency R^2
Lesion	Spatial lesion features	**0.190[0.072;0.289]**/ 0.176[0.079;0.267]	0.000[0.000;0.021]/ **0.057[0.000;0.129]**
Brain Mode	Unicity	0.133[0.053;0.197]	**0.107[0.028;0.170]**
	Equivalence	0.144[0.056;0.209]	0.061[0.000;0.122]
	Association	0.118[0.038;0.192]	0.101[0.018;0.159]
	Inhibition	0.131[0.044;0.197]	0.059[0.000;0.122]
	Summation	**0.169[0.080;0.233]**	0.086[0.001;0.157]
Structural disconnection	Tract-wise disconnection	**0.194[0.117;0.266]**	**0.113[0.037;0.172]**
	Interregional disconnectome	0.136[0.031;0.217]/ 0.118[0.034;0.189]	0.000[0.000;0.047]/ 0.000[0.000;0.060]
	Binary interregional connectome	0.186[0.087;0.282]	0.000[0.000;0.073]
Graph	Δdegree	0.184[0.091;0.252]	0.115[0.025;0.174]
	Δclustering coefficient	0.125[0.036;0.197]	0.104[0.019;0.164]
	Δrich club coefficient	0.050[0.000;0.133]	0.093[0.012;0.163]
	Δdistance	0.176[0.074;0.270]	0.007[0.000;0.083]
	Binary Δdistance	0.121[0.021;0.205]	0.000[0.000;0.000]
	Global efficiency	0.155[0.070;0.211]	0.102[0.008;0.167]

	Characteristic path length	0.184[0.101;0.239]	0.108[0.028;0.169]
	Betweeness centrality	**0.188[0.105;0.257]**	**0.116[0.030;0.178]**

Supplementary Table 6: Results of the control analysis when excluding patients with an mRS=6

Results for the repeated classification modelling with a sample of 726 patients while excluding any patients with an mRS=6 across 50 repetitions. Mean ± Standard Deviation is shown.

Poor vs. favourable functional Outcome (mRS 3 months; mRS=6 excluded)

**Category**	**Measure**	**Prediction Accuracy %**
**Lesion**	Spatial lesion features	66.63 ± 4.26
**Brain Mode**	Summation	66.23 ± 3.27
**Disconnection**	Interregional disconnectome	62.81 ± 3.62
**Graph**	Binary Δdistance	61.48 ± 3.86

Supplementary Table 7: Results of the control analysis with other criteria to define an existing connection in the binary interregional connectome

The main analyses used a definition of $N_{Streamlines}$ = 1 to identify existing connections. To evaluate the potential impact of this criterion, we replicated the prediction analyses with the repeated cross-validation design with 250 repetitions for the binary interregional connectome with $N_{Streamlines}$ = 1,50,100, and 350. Mean ± Standard Deviation is shown.

Streamline criterion	N = 1 (original)	N = 50	N = 100	N = 350
**NIHSS 24h R^2**	0.359 ± 0.073	0.358 ± 0.065	0.357 ± 0.064	0.271 ± 0.071
**mRS 3 months accuracy in %**	62.0 ± 3.8	0.62 ± 3.8	0.61 ± 3.9	0.59 ± 3.7
